# Epidemic and control of COVID-19 in Niger: quantitative analyses in a least developed country

**DOI:** 10.7189/jogh.10.020513

**Published:** 2020-12

**Authors:** Ali Issakou Malam Tchole, Zhen-Wei Li, Jia-Te Wei, Run-Ze Ye, Wen-Jing Wang, Wan-Yu Du, Hai-Tao Wang, Chao-Nan Yin, Xiao-Kang Ji, Fu-Zhong Xue, Alassan Maman Bachir, Lin Zhao, Wu-Chun Cao, Cheeloo EcoHealth Consortium (CLEC)

**Affiliations:** 1 Institute of EcoHealth, School of Public Health, Cheeloo College of Medicine, Shandong University, Jinan, P. R. China; 2 Directorate of Surveillance and Response to Epidemics, Ministry of Public Health, Niamey, Niger; 3 State Key Laboratory of Pathogen and Biosecurity, Beijing Institute of Microbiology and Epidemiology, Beijing, P. R. China; 4 Institute for Medical Dataology, School of Public Health, Cheeloo College of Medicine, Shandong University, Jinan, P. R. China

## Abstract

**Background:**

The COVID-19 pandemic is challenging the public health response system worldwide, especially in poverty-stricken, war-torn, and least developed countries (LDCs).

**Methods:**

We characterized the epidemiological features and spread dynamics of COVID-19 in Niger, quantified the effective reproduction number (*R_t_*), evaluated the impact of public health control measures, and estimated the disease burden.

**Results:**

As of 4 July 2020, COVID-19 has affected 29 communes of Niger with 1093 confirmed cases, among whom 741 (67.8%) were males. Of them 89 cases died, resulting in a case fatality rate (CFR) of 8.1%. Both attack rates and CFRs were increased with age (*P* <  0.0001). Health care workers accounted for 12.8% cases. Among the reported cases, 39.3% were isolated and treated at home, and 42.3% were asymptomatic. 74.6% cases were clustered in Niamey, the capital of Niger. The *R_t_* fluctuated in correlation to control measures at different outbreak stages. After the authorities initiated the national response and implemented the strictest control measures, *R_t_* quickly dropped to below the epidemic threshold (<1), and maintained low level afterward. The national disability-adjusted life years attributable to COVID-19 was 1267.38 years in total, of which years of life lost accounted for over 99.1%.

**Conclusions:**

Classic public health control measures such as prohibition of public gatherings, travelling ban, contact tracing, and isolation and quarantine at home, are proved to be effective to contain the outbreak in Niger, and provide guidance for controlling the ongoing COVID-19 pandemic in LDCs.

The ongoing coronavirus disease 2019 (COVID-19), first identified in Wuhan, China [1], has spread rapidly to all continents, with over 18 million reported cases and 709 511 deaths [2]. The COVID-19 pandemic was not only a public health, but also a socioeconomic crisis [3]. Compared to other regions, the pandemic affected Africa later due to the limited international air traffic [4]. However, as of 7 August 2020, Africa has reported over one million cases across all 54 countries, and nearly two-thirds were experiencing community transmission [5,6]. The figures are likely to underestimate the spread of the virus, because of its relatively low testing capacity and inadequate case reporting system [7,8]. Africa might become the latest epicenter of the pandemic, given the continuously increasing number of cases and the vulnerable health care systems [9].

COVID-19 is presently building up a foothold in poverty-stricken, war-torn, and least developed countries (LDCs) [10], bringing a growing call to identify locally fitted solutions [11]. Niger, the largest country in West Africa, is one of the LDCs, with a vulnerable economy, low literacy, lack of infrastructure, and little access to health care [12]. Niger is presently facing serious threats from armed attacks and abductions [13], which undoubtedly aggravated the health crisis. On 19 March 2020, a 36-year-old Nigerian warehouseman, who worked for a transportation company and had recently traveled overseas, was confirmed as the first COVID-19 case in Niger [14]. Thereafter, the COVID-19 quickly spread throughout the country. Additionally, the migratory population further accelerated the cross-nation and cross-region transmissions. The epidemiological situation of COVID-19 in Niger was complicated, given the social, economic, cultural, and religious realities. This study aimed to investigate the epidemiological characteristics and transmission dynamics of COVID-19 in Niger, evaluate the effects of control measures, estimate the burden of COVID-19, and thus to devise targeted prevention and control efforts and provide evidence-based guidance to LDCs for fighting the current outbreak.

## METHODS

### Data collection and case definition

We obtained data on COVID-19 from the Directorate of Surveillance and Response to Epidemics at the Ministry of Public Health (MoH) of Niger. The number of confirmed cases and death toll from COVID-19 was collected from 19 March to 4 July, 2020. Demographic characteristics (age, gender, occupation, and residence location), epidemiologic characteristics (travel and exposure history, date of symptom onset, date of hospital admission, date of diagnosis, and date of report), and clinical characteristics (medical management, signs and symptoms, and clinical outcomes) of each case were extracted. The available-case method was used for handling the missing values in the age variable [15] and cases with missing information on occupation were identified as “unknown” in the analyses. Confirmed cases were patients who had related epidemiological history and clinical manifestations, and a positive result on real-time reverse transcription polymerase chain reaction (RT-PCR) assay for SARS-CoV-2, conducted by the Center for Medical Research and Health of Niger.

Data regarding control measures deployed in Niger were collected from the website of MoH (https://www.coronavirus.ne). Population data by communes and age groups were derived from the Statistic Yearbook of Niger (http://www.stat-niger.org/frame/index.htm) and the United Nations Department of Economic and Social Affairs (https://population.un.org/wpp/Download/Standard/Population/), respectively. This study was approved by the Ethics Committee of the Directorate of Surveillance and Response to Epidemics at the MoH of Niger (No. 000032). All information regarding individual persons had been anonymized.

### Statistical analysis

The continuous variables were expressed as median (interquartile range, IQR) while the categorical variables were reported as frequency (n) or proportion (%). Differences between groups were tested by χ^2^ test. Attack rate (AR) was calculated by the number of cumulative confirmed cases divided by the population size. Case fatality rate (CFR) was presented as percentage of deaths among identified confirmed cases. These estimates have been based on the COVID-19 data as of 4 July 2020. All statistical analyses were conducted with R software (version 4.0.2) and SPSS software (version 21.0, SPSS Inc, Chicago IL, USA). A two-sided p value less than 0.05 was considered statistically significant.

ArcGIS 10.4 software (ESRI Inc, Redlands CA, USA) was used to prepare a thematic map of commune-level AR. Kulldorff’s purely spatial scan statistics was used to explore the spatial clusters of COVID-19 in Niger [16]. The log likelihood ratio (LLR) was mainly applied to determine the most likely cluster and secondary likely cluster [17]. The relative risk (RR) was a crucial indicator to assess the risk of each cluster, calculated by the estimated risk within the cluster divided by the estimated risk outside the cluster. The number of Monte Carlo simulation was limited to 999 and the statistically significant level was set as 0.05 [16,17]. The spatial cluster analysis was completed using SaTScan^TM^ software (version 9.4, Kulldorff M and Information Management Systems Inc, Boston MA, USA), and visualized by ArcGIS software.

We tracked the effective reproduction number on day t (*R_t_*) in relation to control measures and events. *R_t_* was defined as the mean number of secondary cases generated by one primary case with symptom onset on day t, which was estimated using the method developed by Cori et al. (Text S1 in the **Online Supplementary Document**) [18]. Confidence intervals (CI) were quantified using a bootstrap procedure [19].

### Estimating the burden of COVID-19

The burden of COVID-19 in Niger was estimated by disability-adjusted life years (DALYs), which combines the years of life lost (YLLs) due to premature mortality and years lived with disability (YLDs) [20]. DALYs were calculated for different gender and age categories, using the following formulas [21]:


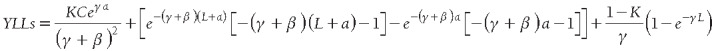
(1)


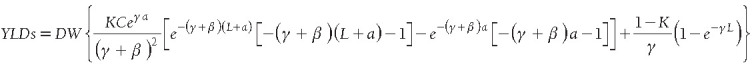
(2)

Where *K* is the age weighting modulation constant, and *C* is the adjustment constant for age-weights; *e* represents the life expectancy of each age group; γ is the discount rate and β is the age weighting constant [21-23]. Values of the above parameters were based on the Global Burden of Disease template provided by the World Health Organization (WHO) [22]. *L*, defined as the duration of COVID-19, was set to 28 days [24]. *DW*, the disability weight, is an essential weight factor reflecting the disease severity between 0 (full health) and 1 (death). Since no *DW* standard is available yet for COVID-19, it was set to 0.133 according to lower respiratory tract infection, whose health outcome is comparable to COVID-19 [21]. The parameter *a* was the average age of death for YLLs calculation and the average age of onset for YLDs.

## RESULTS

As of 4 July 2020, 1093 confirmed cases had been reported in Niger (**Table 1**), with a median age of 43 years (IQR = 28-57 years). Males were twice as likely to be infected than females (67.8% vs 32.2%). Health care workers accounted for 12.8% of the confirmed cases, followed by students (9.8%), housewives (6.9%), and soldiers (4.2%). The time intervals between symptom onset to hospitalization, diagnosis, and report were 3 (IQR = 1-7 days), 5 (IQR = 2-9 days), and 6 days (IQR = 3-10 days), respectively. 62 imported cases were Nigerians who had returned mainly from the neighboring countries (such as Ghana, Nigeria, Senegal, and Mali) and some European countries (such as France, Turkey, and Switzerland) (Figure S1 in the **Online Supplementary Document**). Given the relatively stable health conditions and limited health care resources, 39.3% cases were isolated and treated at home. Among the reported cases, 42.3% were asymptomatic. The clinical characteristics of the 631 symptomatic cases are summarized in **Table 1**. Cough (69.9%) and fever (63.2%) were the most prevalent symptoms, followed by respiratory symptoms, such as shortness of breath (36.9%), sore throat (23.6%), runny nose (18.2%), and chest pain (9.4%). Systemic symptoms, involving headache (15.7%), fatigue (9.0%), anosmia (7.8%), and malaise (2.2%) also presented. A few cases had experienced gastrointestinal symptoms, including diarrhea (2.7%) and anorexia (2.2%). As of 4 July 2020, 899 (82.3%) patients had recovered from COVID-19, whereas 105 (9.6%) remained in the hospital under treatment.

**Table 1:** Demographic, epidemiologic, and clinical characteristics of COVID-19 cases in Niger*

Characteristics	COVID-19 cases (n = 1093)
**Date of report**	19 March 2020 – 4 July 2020
**Age (years)†**	43 (28-57)
**Gender:**	
Male	741 (67.8%)
Female	352 (32.2%)
**Occupation:**	
Health care workers	140 (12.8%)
Soldiers	46 (4.2%)
Students	107 (9.8%)
Housewives	75 (6.9%)
Others	537 (49.1%)
Unknown	188 (17.2%)
**Interval between symptom onset and hospitalization (days)**	3 (1-7)
**Interval between symptom onset and diagnosis (days)**	5 (2-9)
**Interval between symptom onset and report (days)**	6 (3-10)
**Travelling overseas during the last 21 d:**	
Yes	62 (5.7%)
No	1031 (94.3%)
**Patient care model:**	
Treatment in a hospital	663 (60.7%)
Treatment at home	430 (39.3%)
**Signs and symptoms:**	
Asymptomatic case	462 (42.3%)
Symptomatic case:	631 (57.7%)
Cough	441 (69.9%)
Fever	399 (63.2%)
Shortness of breath	233 (36.9%)
Sore throat	149 (23.6%)
Runny nose	115 (18.2%)
Chest pain	59 (9.4%)
Headache	99 (15.7%)
Fatigue	57 (9.0%)
Anosmia	49 (7.8%)
Malaise	14 (2.2%)
Diarrhea	17 (2.7%)
Anorexia	14 (2.2%)
**Clinical outcome:**	
Remained in hospital	105 (9.6%)
Discharged	899 (82.3%)
Died	89 (8.1%)

*Data are median (IQR), n (%), or n/N (%).

†11 (1.0%) cases with missing values have been deleted when calculating.

**Figure 1:**
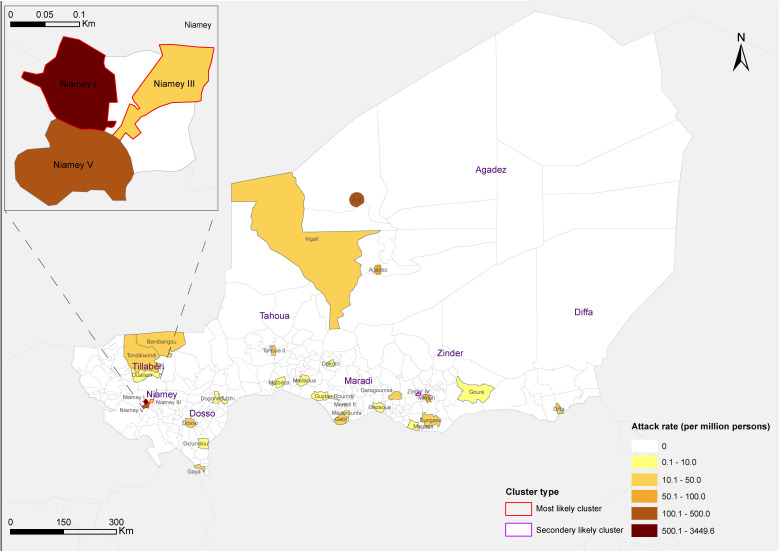
Geographical distribution and spatial clustering of COVID-19 cases in Niger. Thematic map shows the commune-level attack rate of COVID-19 in Niger. Areas highlighted with red or pink edging represent most likely cluster area and secondary likely cluster area, respectively.

**Figure 1** shows the geographical distribution and spatial clustering of COVID-19 cases in Niger. COVID-19 has affected 29 communes in all 8 regions (7 regions and the capital district). Although small in size, nearly three-fourths of confirmed cases were reported in Niamey, with an overall AR of 1078.3 per million persons. Communes with ARs over 100.0 per million persons were dispersed in Niamey I (3449.6 per million persons), Niamey V (204.4 per million persons), Arlit (438.0 per million persons), and Zinder IV (343.7 per million persons). About half of the affected communes had ARs lower than 15.0 per million persons. According to the spatial clustering analysis, the most likely spatial cluster area was located in Niamey I and Niamey III communes (LLR = 1512.5, RR = 36.7, *P* < 0.0001), and the secondary likely cluster was distributed in Zinder commune (LLR = 46.1, RR = 3.0, *P* < 0.0001). COVID-19 cases in the above three communes accounted for 81.1% of the total cases in Niger.

**Figure 2:**
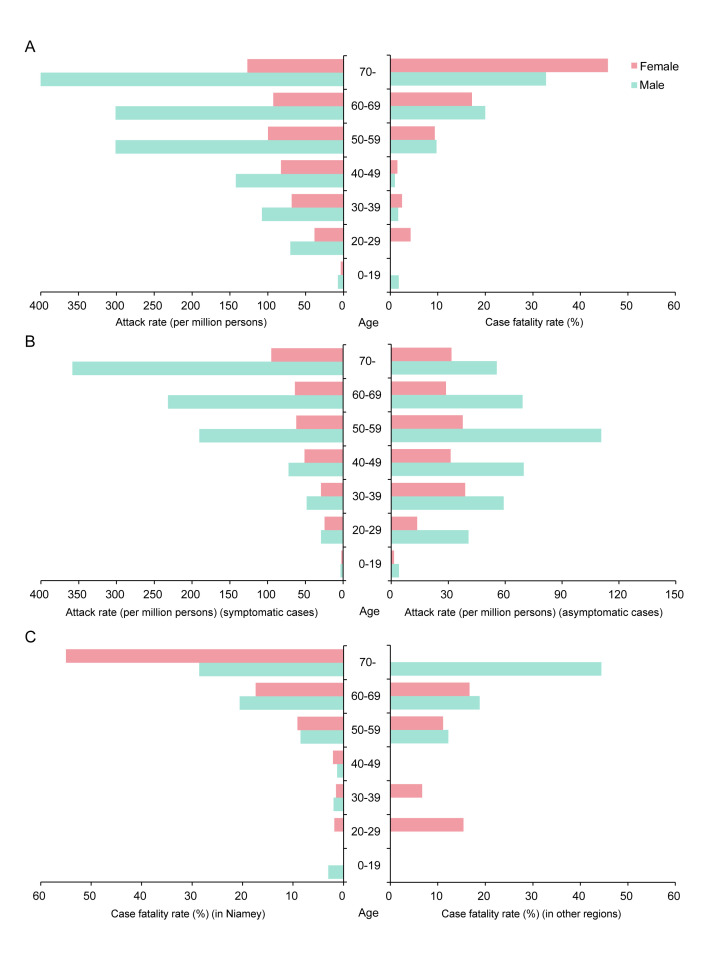
Age and gender differences in incidence and fatality of COVID-19 in Niger. **Panel A.** The attack rate (AR) and case fatality rate (CFR). **Panel B.** AR of symptomatic cases (left) and asymptomatic cases (right). **Panel C.** CFR in Niamey (left) and other regions (right).

As shown in **Figure 2**, Panel A, the overall AR of COVID-19 in the male population (60.9 per million persons) was significantly higher than the female (29.2 per million persons, *P* < 0.0001). Gender differences were found to be significant across all age categories (all *P* < 0.05). As age increased, the ARs were increased significantly (χ^2^ test for trend, *P* < 0.0001), with individuals over 60 years old having 14 times higher than those younger than 30 years (221.0 vs 15.6 per million persons, *P* < 0.0001). The overall CFR of COVID-19 was 8.1% (89/1093) in Niger, with no significant difference between males (8.4%) and females (7.7%, *P* = 0.735) (**Figure 2**, Panel A). The CFRs increased strongly with age in both genders (χ^2^ test for trend, *P* < 0.0001), which was 19 times higher among cases over 60 years compared with those under 30 years (26.4% vs 1.4%, *P* < 0.0001). The male and female differences in ARs were significant in both the symptomatic case group and the asymptomatic group (**Figure 2**, Panel B). For symptomatic cases, the ARs in both genders increased significantly with age (χ^2^ test for trend, *P* < 0.0001). For asymptomatic cases, individuals in the age group of 50-59 years had the highest AR (73.5 per million persons), and there were no significant differences in ARs between people older than 60 years (47.2 per million persons) and aged 20 to 49 years (38.4 per million persons, *P* = 0.217). **Figure 2**, Panel C, demonstrates that the CFR of COVID-19 in Niamey, the epicenter (7.9%), was not significantly different from that in other regions (9.0%, *P* = 0.548). The CFRs showed no significant differences between genders either in Niamey (7.8% for male vs 8.0% for female, *P* = 0.911), or in other regions (10.0% for male vs 6.5% for female, *P* = 0.367). The increasing trend of CFRs with advancing age remained consistently across different regions of Niger (χ^2^ test for trend, both *P* < 0.0001).

**Figure 3:**
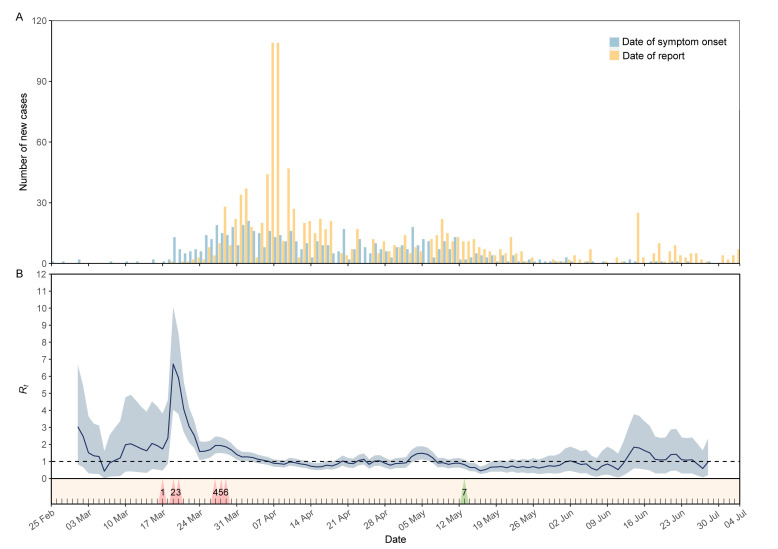
Epidemic curve and estimated effective reproduction number (*R_t_*) during the COVID-19 outbreak in relation to public health control measures in Niger. **Panel A.** Epidemic curve (symptom onset and report date) of cases. **Panel B.** Estimated *R_t_* in relation to public health control measures. Values represent average *R_t_* (central dark blue line) and associated 95% confidence interval (light blue shade), by date of symptom onset. The critical value of *R_t_* = 1 is marked with a horizontal dot line, below which sustained transmission is impossible. Triangles at the bottom represent the moment of important public health control measures: (1) The practice of social distancing in Niamey (17 March 2020); (2) The Ministry of Public Health announced the first COVID-19 case in Niger, and the authorities developed the national COVID-19 Emergency Preparedness and Response Plan (19 March 2020); (3) Targeted control measures have been implemented in Niamey and throughout the country, such as closure of places of worship, health screenings and border control, banned on non-essential visits to remand centers, quarantine at home and establishment of temporary hospitals (20 March 2020); (4) The authorities declared the state health emergency (27 March 2020); (5) Travel ban and Curfew in Niamey (28 March 2020); (6) Lockdown of Niamey (29 March 2020); (7) Reopened places of worship (13 May 2020).

The outbreak in Niger started on 25 February and peaked around 2 April 2020 (**Figure 3**, Panel A). The majority of cases were diagnosed and reported between 6-11 April 2020. Thereafter, the number of new cases decreased dramatically and remained at a relatively low level. The COVID-19 response in Niger has been progressive and swift, despite having limited resources. Before the first case was identified, the practice of social distancing, including the closure of schools and prohibition of public gatherings (Figure S2 in the **Online Supplementary Document** for detail), had been adopted in Niger on 17 March 2020 (triangle 1 in **Figure 3**, Panel B). The *R_t_* fluctuated between 0.4 and 3.0 with a wide 95% CI, when the virus silently occurred in Niger. On 19 March, the authorities developed the national COVID-19 Emergency Preparedness and Response Plan to effectively respond to the outbreak of COVID-19 (triangle 2). However, the *R_t_* sharply increased to the peak value of 6.7 due to the rapid spread of virus within Niamey and to other regions. Subsequently, a series of targeted control measures were implemented on 20 March (triangle 3), such as closure of places of worship, health screenings and border control, banned on non-essential visits to remand centers, establishment of temporary hospitals and quarantine at home. Immediately, a considerable decrease of *R_t_* was noticed. One week later, Niger declared the state health emergency (triangle 4), and started travel ban and curfew in Niamey (triangle 5). Considering the high exportations and spread risk, Niamey was locked down on 29 March (triangle 6). Thereafter, the *R_t_* maintained downward to below one within a week (on 7 April). Since then, *R_t_* fluctuated slightly around one, even if Niger reopened the places of worship on 13 May (triangle 7).

During the study period, the DALYs attributable to COVID-19 were 1267.38 years in total, and 0.05 years per 1000 population in Niger. The disease burden mainly came from people over 45 years old (82.59% of total DALYs), and males experienced a higher burden than females (832.96 vs 434.42). For both genders, adults in the 45-59 years age group had the highest DALYs, while boys aged 5-14 years and girls aged 0-4 years had the lowest. People older than 80 years had the highest DALYs per 1000 population, with 1.78 in males and 1.21 in females. Overall, mortality dominated the disease burden, since YLLs accounted for over 99.1% of DALYs. The only exception was in males aged 5-29 years and females aged 5-14 years, where YLDs was the major component of DALYs. Additionally, the highest YLDs appeared in different age groups, with 2.19 in males aged 45-59 years and 1.24 in females aged 30-44 years (**Table 2**).

**Table 2:** Disability-adjusted life years (DALYs) of COVID-19 in Niger

Age Group	Males						Females						Total population			
**YLLs**	**YLDs**	**DALYs**	**DALYs per 1000 males**	**% of YLLs in DALYs**	**% of total DALYs**	**YLLs**	**YLDs**	**DALYs**	**DALYs per 1000 females**	**% of YLLs in DALYs**	**% of total DALYs**	**DALYs**	**DALYs per 1000 population**	**% of YLLs in DALYs**	**% of total DALYs**
0-4	30.25	0.02	30.27	0.01	99.93	3.63	0.00	0.00	0.00	0.00	NA	0.00	30.27	0.01	99.93	2.39
5-14	0.00	0.24	0.24	0.00	0.00	0.03	0.00	0.13	0.13	0.00	0.00	0.03	0.37	0.00	0.00	0.03
15-29	0.00	1.70	1.70	0.00	0.00	0.20	82.35	0.88	83.23	0.03	98.94	19.16	84.93	0.01	96.96	6.70
30-44	48.90	1.68	50.58	0.03	96.68	6.07	53.18	1.24	54.42	0.03	97.72	12.53	105.00	0.03	97.22	8.28
45-59	293.73	2.19	295.92	0.35	99.26	35.53	120.28	0.83	121.11	0.14	99.31	27.88	417.03	0.24	99.28	32.90
60-69	278.94	1.03	279.97	0.84	99.63	33.61	75.91	0.30	76.21	0.24	99.61	17.54	356.18	0.55	99.63	28.10
70-79	132.41	0.49	132.90	0.96	99.63	15.96	60.84	0.16	61.00	0.39	99.74	14.04	193.90	0.66	99.66	15.30
≥80	41.17	0.21	41.38	1.78	99.49	4.97	38.23	0.09	38.32	1.21	99.77	8.82	79.70	1.45	99.62	6.29
Total	825.40	7.56	832.96	0.07	99.09	100.00	430.79	3.63	434.42	0.04	99.16	100.00	1267.38	0.05	99.12	100.00

YLLs – years of life lost, YLDs – years lived with disability, NA – not applicable

## DISCUSSION

COVID-19 pandemic poses great challenges for maintaining global health security, and has caused great concern about unprecedented health crisis especially in poverty-stricken, war-torn LDCs, where both health care and economic systems are particularly vulnerable [9-11]. Our findings on the epidemic trajectory of COVID-19 in Niger indicate that the outbreak can be curtailed even in LDCs, as long as collective interventions are effectively implemented. All infection control measures should be properly undertaken with the aim of modulating the epidemic trajectory so that the epidemic wave does not overwhelm local health care system capabilities [25].

Our findings suggest that the male cases outnumbered the females, which is disparate from those in mainland China and Korea [26,27]. One possible reason for a higher infection risk among males in Niger is due to the local cultural situation. Males have to be engaged in communal activities to earn money for their families even during the outbreak, while females tend to stay at home. In addition, men in Niger have more access to the places of worship and congregations, which subsequently increases the risk of infection. Both AR and CFR significantly increased with age, given a younger age distribution of Niger [28]. Cumulative evidence indicated that males infected with COVID-19 had higher mortality than females, due to the differences inimmunological background and lifestyle, such as smoking and drinking [26]. Compared with symptomatic cases, asymptomatic cases were largely reported in the younger age groups, who are healthier and have fewer ACE2 receptors [29].

In Niger, the shortage of health care resources resulted in about 40% of cases receiving isolation and treatment at home. Despite this, the proportion of infected health care workers is up to 12.8%, which is much higher than other countries [30]. The main reason is lack of personal protection equipment (PPE) for health care workers on the front line. Providing adequate PPE and enhancing infection control measures within hospitals are essential in Niger as well as other LDCs not only to protect health care workers and preserve the health care system, but also to prevent nosocomial infections that might eventually foster a larger community transmission. After WHO declared COVID-19 as a pandemic, many Nigerians who left for a rural exodus, trade, business, or even diplomatic missions overseas decided to return. As a result, some returned Nigerians constitute the infective sources of COVID-19. The analysis of geographical distribution and spatial clustering revealed an epicenter in capital Niamey, where the majority of infected people and their close contacts were imported. The convenient transportation system and more crowded population might have facilitated the virus transmission. Surely, an undeniable fact is that the people in the capital city have more opportunities to get laboratory tests, and thus are more likely to be diagnosed. Anyway, the strict public health control measures, including lockdown of Niamey, turn to be effective to control the dissemination of COVID-19 from Niamey to other regions. The regions of Zinder and Agadez constitute the other two most affected areas after Niamey. Agadez region is the transit area for migrants with high population flow. The high AR of Zinder is probably caused by the frequent trade with Nigeria, one of the countries most affected by COVID-19 in West Africa; and also by the influx of refugees from Diffa and Maiduguri.

*R_t_* reflects the transmissibility of SARS-CoV-2 at different times, and can be used to simulate scenarios for different interventions to determine whether additional control measures are needed [18]. In Niger, efforts to mitigate the spread of COVID-19 began early. Before the first case was identified, guidance for preventing COVID-19 was announced to the public, with emphasis on hand hygiene and social distancing. On 19 March, when the first case was confirmed, Nigerian authorities initiated the national response and put into place the most comprehensive and rigorous measures. However, since many people neither believe the disease exists in the country nor comply with protection standards, Nigerian authorities announced the state health emergency on 27 March, and continued to strengthen prevention and control measures in the following days. Since then, the Nigerians were more aware of the COVID-19 pandemic and how to protect themselves and families. Within a week, *R_t_* dropped to below the threshold value of one. After May, Niger eased some COVID-19 restrictions, but *R_t_* has never increased dramatically again. These facts imply that trust between people and authorities should be maintained so that communities and individuals adhere to the medical advice by institutions [25], and classic public health control measures, such as prohibition of public gatherings, travelling ban, contact tracing, and isolation and quarantine at home can actually contain the disease spread. However, these measures should be implemented with prudence while considering their cost efficiency [25]. For an LDC, proactively striking a balance between keeping the local economic alive and keeping *R_t_* below one is likely to be the optimal strategy until effective vaccines and antiviral drugs become widely available, despite the fact that collective non-pharmaceutical interventions will probably be maintained for some time [31].

At present, there’s a lack of research that deals with the disease burden of COVID-19, and none of them focuses on the LDCs [23,27]. People aged over 45 years contributed to most of the total DALYs, suggesting that elderly people with more pre-existing comorbidities tended to experience a higher risk of COVID-19. The associations between COVID-19 severity, mortality and pre-existing diseases have been described by previous studies [32]. The DALYs per 1000 population were higher in males than in females and increased with age, which coincided with the trend in Italy [23]. The gender- and age-wise distribution of DALYs was consistent with that of ARs in Niger. The vast majority of the disease burden is attributed to early mortality, with YLLs accounting for over 99% of DALYs, suggesting the importance of reducing fatality of COVID-19 by improving the accessibility and quality of health care services in Niger. It should be pointed out that long-term impact on disability caused by COVID-19 needs further investigation, and the proportion of YLDs should be underestimated because of the short study period.

There are some limitations of this study. First, the lack of laboratory tests might have created delays in identifying cases, and the number of reported cases might be underestimated. Second, the health care resources were disproportionately distributed across Niger, and the public awareness of COVID-19 in distant areas was relatively low. Therefore, cases in remote communes with limited resources were more likely to be underreported. Third, there were 105 cases remaining in hospitals by the time of this reporting, whose clinical outcomes remained unknown. CFR and DALYs in Niger might have been underestimated.

## CONCLUSIONS

In conclusion, the ongoing COVID-19 outbreak spread to a wide range in Niger. National response and public health control measures have shown obvious effects on restraining the epidemic areas and slowing down the transmission. Though the future evolution of this outbreak remains unpredictable, classic public health strategies deployed in Niger based on the local social-economic and cultural settings should provide optimal guidance for other LDCs to effectively fight against the ongoing pandemic of COVID-19.

## Additional material

Online Supplementary Document
