# Supplementary material for: Epidemic and control of COVID-19 in Niger: quantitative analyses in a least developed country
Source: J Glob Health. 2020 Dec 6;10(2):020513. doi: 10.7189/jogh.10.020513 (PMC7719275; doi:10.7189/jogh.10.020513)
Supplement: Online Supplementary Document [file jogh-10-020513-s001.pdf]

Supplementary Text S1. Detailed method of estimating effective reproduction number (R_t).

Basic reproduction number vs. time-varying reproduction numbers

The key epidemiologic variable that characterizes the transmission potential of a disease is the basic reproduction number, R_0, which is defined as the average number of secondary infections produced by a typical case of an infection in a population where everyone is susceptible. However, the basic reproduction number is affected by the duration of infectiousness. The basic regeneration during an outbreak is not accurate and does not reflect the instantaneous state of disease transmission well. Therefore, the time-varying reproduction numbers during epidemics are more concerned.

The monitoring of R over time provides feedback on the effectiveness of control measures and depletion of susceptible persons during the epidemic. If R_t exceeds 1, the number of cases will inevitably increase over time, and a large epidemic is possible. To stop an epidemic, the goal of intensify control efforts is to reduce R_t below the threshold value of 1 and as close to 0 as possible, thus bringing an epidemic under control. Moreover, effective control measures undertaken at time t are expected to result in a sudden decrease in R_t. Hence, assessing the efficiency of control measures is easier by using estimates of R_t.

Method to estimate R_t

It is assumed that once infected, an individual had an infectivity profile given by the probability distribution ϖ_s that depends on the time s after the case was infected, but not on the calendar time t. In other words, individuals become infectious from the moment they are infected, and become more so over time. When ϖ_s reaches its maximum, individuals are most infectious at time s and then the infectivity gradually decreasing. The distribution of ϖ_s usually depends on a single biological factor, such as the shedding of pathogens or the severity of symptoms.

Modelling transmission with a Poisson process, the rate at which someone infected in time step t-s generates new infections in time step t, is equal to $R_t\varpi_s$, where R_t is the instantaneous reproduction number at time t and ϖ_s is the probability distribution of the average infectiousness profile after infection. Given the definition of R_t stated above,

the incidence at time step t is Poisson distributed with mean $R_t \sum_{s=1}^{t} I_{t-s}\varpi_s$,

$$I_t \sim Po(R_t \sum_{s=1}^{t} I_{t-s}\varpi_s)$$

Thus, the likelihood of the incidence at t, conditional on the previous incidences is:

$$P(I_t \mid I_0,\ldots,I_{t-1},\varpi,R_t) = \frac{(R_t \sum_{s=1}^{t} I_{t-s}\varpi_s)^{I_t} \times e^{-R_t \sum_{s=1}^{t} I_{t-s}\varpi_s}}{I_t!}$$

If the rate of transmission is assumed constant over a time period $[t-\tau+1,t]$ (a time window of size τ ending at time t), the likelihood of the incidence during this time period is

$$P(I_{t-\tau+1},\ldots,I_t \mid I_0,\ldots,I_{t-\tau},\varpi,R_{t,\tau}) = \prod_{s=t-\tau+1}^{t} \frac{(R_{t,\tau} \sum_{q=1}^{s} I_{s-q}\varpi_q)^{I_s} \times e^{-R_{t,\tau} \sum_{q=1}^{s} I_{s-q}\varpi_q}}{I_s!}$$

Applying a Bayesian framework with a Gamma distributed prior with parameters (a,b) , the

posterior joint distribution of $R_{t,\tau}$ is

$$\begin{aligned}
&P(I_{t-\tau+1},\ldots,I_t \mid I_0,\ldots,I_{t-\tau},\varpi) \\
&= P(I_{t-\tau+1},\ldots,I_t \mid I_0,\ldots,I_{t-\tau},\varpi,R_{t,\tau})P(R_{t,\tau}) \\
&= \left(\prod_{s=t-\tau+1}^{t} \frac{(R_{t,\tau} \sum_{q=1}^{s} I_{s-q}\varpi_q)^{I_s} \times e^{-R_{t,\tau} \sum_{q=1}^{s} I_{s-q}\varpi_q}}{I_s!} \right) \times \left(\frac{R_{t,\tau}{}^{a-1} e^{\frac{-R_{t,\tau}}{b}}}{\Gamma(a) b^a} \right) \\
&= R_{t,\tau}{}^{a+\sum_{s=t-\tau+1}^{t} I_s - 1} \times e^{-R_{t,\tau}\left(\sum_{s=t-\tau+1}^{t} \sum_{q=1}^{s} I_{s-q}\varpi_q + \frac{1}{b} \right)} \times \prod_{s=t-\tau+1}^{t} \frac{(\sum_{q=1}^{s} I_{s-q}\varpi_q)^{I_s}}{I_s!\Gamma(a) b^a}
\end{aligned}$$

Which is proportional to:

$$R_{t,\tau}^{\,a+\sum_{s=t-\tau+1}^{t} I_s - 1} \times e^{-R_{t,\tau}\left(\sum_{s=t-\tau+1}^{t}\left(\sum_{q=1}^{s} I_{s-q}\varpi_q\right)+\frac{1}{b}\right)} \times \prod_{s=t-\tau+1}^{t} \frac{\left(\sum_{q=1}^{s} I_{s-q}\varpi_q\right)^{I_s}}{I_s!}$$

Therefore, the posterior distribution of $R_{t,\tau}$ is

$$R_{t,\tau} \sim Gamma\left(a + \sum_{s=t-\tau+1}^{t} I_s, \frac{1}{\sum_{s=t-\tau+1}^{t}\sum_{q=1}^{s} I_{s-q}\varpi_q + \frac{1}{b}} \right)$$

Then, this allows to directly obtain any desired posterior distribution characteristics (for example, mean, median, variance, or 95% confidence interval).

Time window τ

These estimates of $R_{t,\tau}$ quantify the average transmissibility over a time window of length τ ending at time t. The value of estimates depends on the choice of time window size τ. When τ is small, the detection of transmission changes is rapid but with more statistical noise; Conversely when t is large, the R_t will be smoother and with less statistical noise, resulting to more precise result.

Uncertainty of infectiousness profile

This estimation approach illustrated above is based on the ideal situation in which times of infection are known and the infectiousness profile w_s could be approximated by the distribution of the generation time (i.e., time from the infection of a primary case to infection of the cases he or she generates). However, times of infection are rarely observed and the generation time distribution is therefore difficult to measure. In order to address this problem, the distribution of the serial interval come into our consideration. Serial interval is defined as the time between symptoms onset of a case and symptoms onset of his/her secondary cases, which can be estimated by the timing of symptoms onset. This is usually known and easily collected.

Therefore, in practice, we apply our method on data consisting of daily counts of symptoms onset and where the infectiousness profile w_s is approximated by the distribution of the serial interval. In this research, the distribution of serial interval of COVID-19 and SARS was assumed to be gamma distribution (for COVID-19: mean valued 4.7 days, SD valued 2.9 days; [1] for SARS: mean valued 8.4 days, SD valued 3.8 days [2]).

Reference

1. Nishiura H, Linton NM, Akhmetzhanov AR. Serial interval of novel coronavirus (COVID-19) infections. Int J Infect Dis **2020**; 93:284-6.

2. Marc Lipsitch, Ted Cohen, Ben Cooper et al. Transmission Dynamics and Control of Severe Acute Respiratory Syndrome. Science **2003**; 300:1966-70.

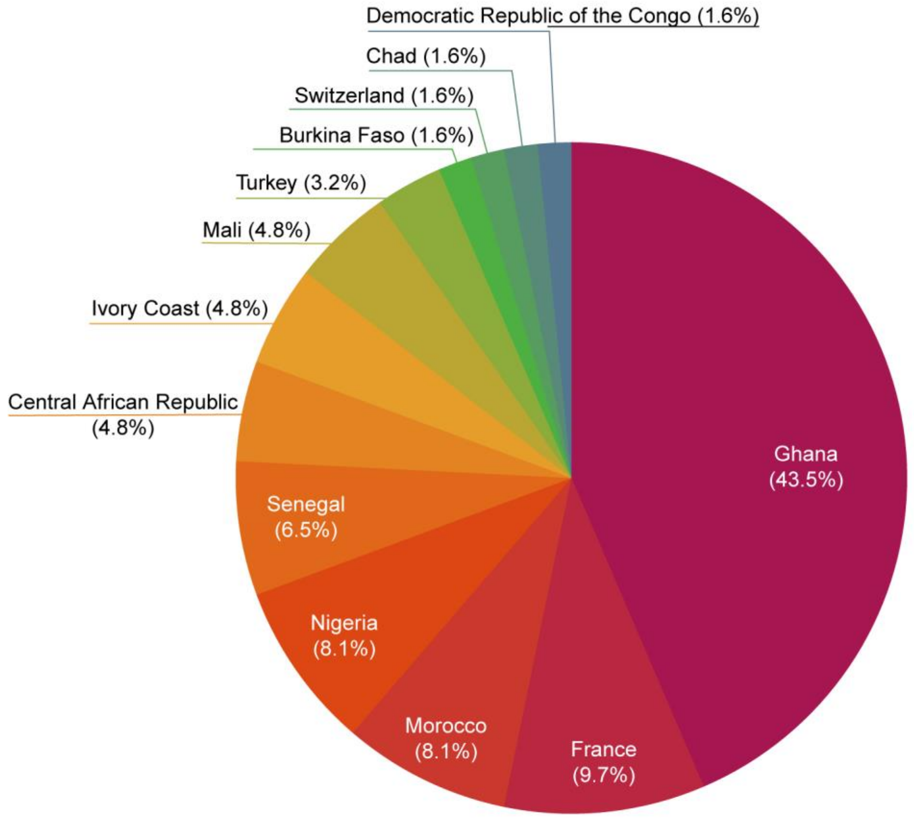

Supplementary Figure S1. Countries where confirmed cases in Niger imported from. Travelling history during the last 21 days before diagnosis of COVID-19.

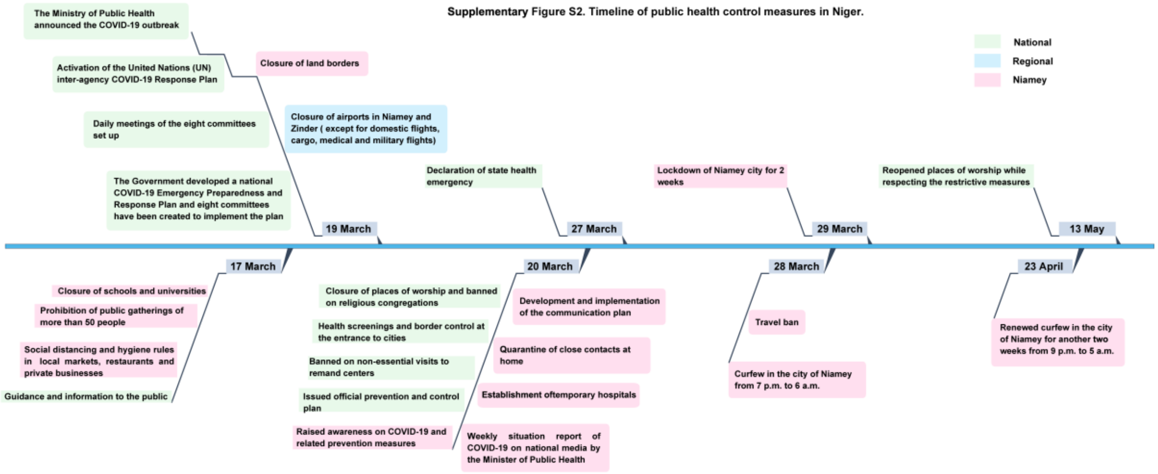

Supplementary Figure S2. Timeline of public health control measures in Niger.
